# An ARG1-associated immunometabolic program in bone marrow PMN-MDSC-like cells in multiple sclerosis and experimental autoimmune encephalomyelitis

**DOI:** 10.3389/fimmu.2026.1924483

**Published:** 2026-10-14

**Authors:** Hengchang Xie, Yiwei Fu, Yingchun Bi, Ranran Han, Pengfei Liu, Bolin He, Baoting Chen, Honglei Ren

**Affiliations:** 1 Department of Neurology, Tianjin Neurological Institute, Laboratory of Post-Neuroinjury Neurorepair and Regeneration in Central Nervous System Tianjin and Ministry of Education, State Key Laboratory of Experimental Hematology, Tianjin Medical University General Hospital, Tianjin, China; 2 Thermoregulation and Inflammation Laboratory, Chengdu Medical College, Chengdu, Sichuan, China; 3 Department of Oncology, Laboratory of Immunity, Inflammation and Cancer, The First Affiliated Hospital of Chongqing Medical University, Chongqing, China

**Keywords:** ARG1, bone marrow, experimental autoimmune encephalomyelitis, multiple sclerosis, neuroinflammation, PMN-MDSC, single-cell RNA sequencing

## Abstract

**Background:**

Multiple sclerosis (MS) is a chronic inflammatory demyelinating disease of the central nervous system. Recent work indicates that bone-marrow hematopoietic niches sense CNS autoimmunity and enhance myeloid output. However, whether MS bone marrow contains an immunometabolic PMN-MDSC-like state, and whether related ARG1-associated observations can be examined in a cross-species EAE context, remains unclear.

**Methods:**

We reanalyzed single-cell RNA sequencing data from treatment-naive patients with MS and healthy controls, focusing on myeloid states. We used donor-aware proportion analysis, trajectory inference, module scoring, donor-pseudobulk pathway enrichment, expression-based transcription-factor prioritization, co-expression analysis, ligand-target inference, and ARG1 virtual perturbation. We also quantified flow-gated bone marrow PMN-MDSCs and ARG1 protein in MOG35-55-induced EAE and tested systemic prophylactic oral nor-NOHA.

**Results:**

MS bone marrow showed myeloid skewing and a heterogeneous PMN-MDSC-like transcriptional state, while donor-level subtype proportions were comparable between groups. Neutrophil degranulation showed the strongest BH-supported donor-pseudobulk enrichment, and expression-based regulatory and communication analyses converged on an ARG1-associated network. In EAE, flow-gated bone-marrow PMN-MDSCs and ARG1 protein were increased, and systemic prophylactic nor-NOHA was associated with lower clinical scores and reduced spinal-cord pathology.

**Conclusions:**

These data identify an ARG1-associated immunometabolic program in bone-marrow PMN-MDSC-like cells and connect the human transcriptional landscape with ARG1 protein expression and pharmacologic responses in EAE. Together, the findings establish ARG1-associated immunometabolism as a focused pathway for mechanistic and translational investigation.

## Introduction

Multiple sclerosis is an autoimmune disease characterized by CNS inflammatory infiltration, demyelination, and axonal injury. Current disease models emphasize interactions among autoreactive T cells, B cells, antigen-presenting cells, and CNS-resident myeloid cells (1–5). These inflammatory cells, however, are not generated only within local CNS lesions. Neutrophils, monocytes, and other short-lived myeloid cells require continuous replenishment from the bone marrow, suggesting that the hematopoietic niche may shape the quantity, state, and inflammatory potential of peripheral myeloid cells entering CNS autoimmune responses.

Recent studies have redefined the relationship between bone marrow and CNS immunity. Skull and vertebral bone marrow can act as reservoirs for myeloid cells in the meninges and CNS parenchyma (6, 7). In MS, Shi et al. reported that hematopoietic stem and progenitor cells (HSPCs) from treatment-naive patients are skewed toward myeloid differentiation, and that enhanced bone marrow myeloid output aggravates CNS inflammation in EAE (8). In neuromyelitis optica spectrum disorder (NMOSD), Li et al. showed aberrant bone marrow granulopoiesis with high JAK-STAT activity and expansion of ISG15+ neutrophils that promote B-cell activation, plasma cell maturation, and AQP4-IgG-associated autoimmunity through BAFF (9). These findings support the concept that bone marrow is not a passive bystander in CNS autoimmune disease. Instead, abnormal granulopoiesis may be a shared upstream process, while downstream effector axes may differ across diseases.

Myeloid-derived suppressor cells (MDSCs) have context-dependent roles in autoimmune disease. In EAE, MDSCs expressing a self-antigen can induce tolerance and ameliorate disease (10). During peptide immunotherapy, PMN-MDSCs acquire a suppressive phenotype and inhibit CD4+ T-cell proliferation through an ARG1-dependent contact mechanism (11). In patients with MS, peripheral blood studies have linked elevated miR-223 to reduced MDSC number, lower STAT3/ARG1 expression, and impaired suppressive function; inhibition of miR-223 or glucocorticoid treatment enhances STAT3-dependent MDSC suppressive activity (12, 13). These studies implicate an MDSC/ARG1/STAT3 axis in MS immune homeostasis, but they mainly focus on blood or experimental tolerance models. Whether PMN-MDSCs expand in the MS bone marrow, whether this state is centered on ARG1, and whether it connects to T-cell suppression and CNS inflammation remain unresolved (14).

We therefore mapped the ARG1-associated PMN-MDSC-like immunometabolic state within granulocytic expansion in MS bone marrow. Human bone-marrow single-cell analyses identified a heterogeneous ARG1-associated granulocytic program; EAE experiments showed increased bone-marrow PMN-MDSCs and ARG1 protein together with disease amelioration after systemic prophylactic arginase-pathway inhibition. These findings connect the human marrow program to a pharmacologically responsive ARG1-associated pathway *in vivo*.

## Results

### Bone marrow single-cell profiling reveals myeloid skewing and a PMN-MDSC-like functional state in MS

We first examined the architecture of the published human bone-marrow single-cell atlas (8). UMAP separated HSPC, erythroid, myeloid, and lymphoid compartments (**Figure 1A**), and the expected lineage markers supported these assignments (**Figure 1B**). CD34, KIT, and SPINK2 localized to HSPC regions; AZU1, MPO, ELANE, CSF3R, LYZ, S100A8, and S100A9 marked myeloid cells; HBA1, HBA2, HBB, and AHSP defined erythroid cells; and CD3D, NKG7, GNLY, MS4A1, CD79A, and MZB1 delineated lymphoid and plasma-cell regions. Thus, the broad atlas provided a consistent framework for resolving disease-associated changes within the myeloid lineage.

**Figure 1:**
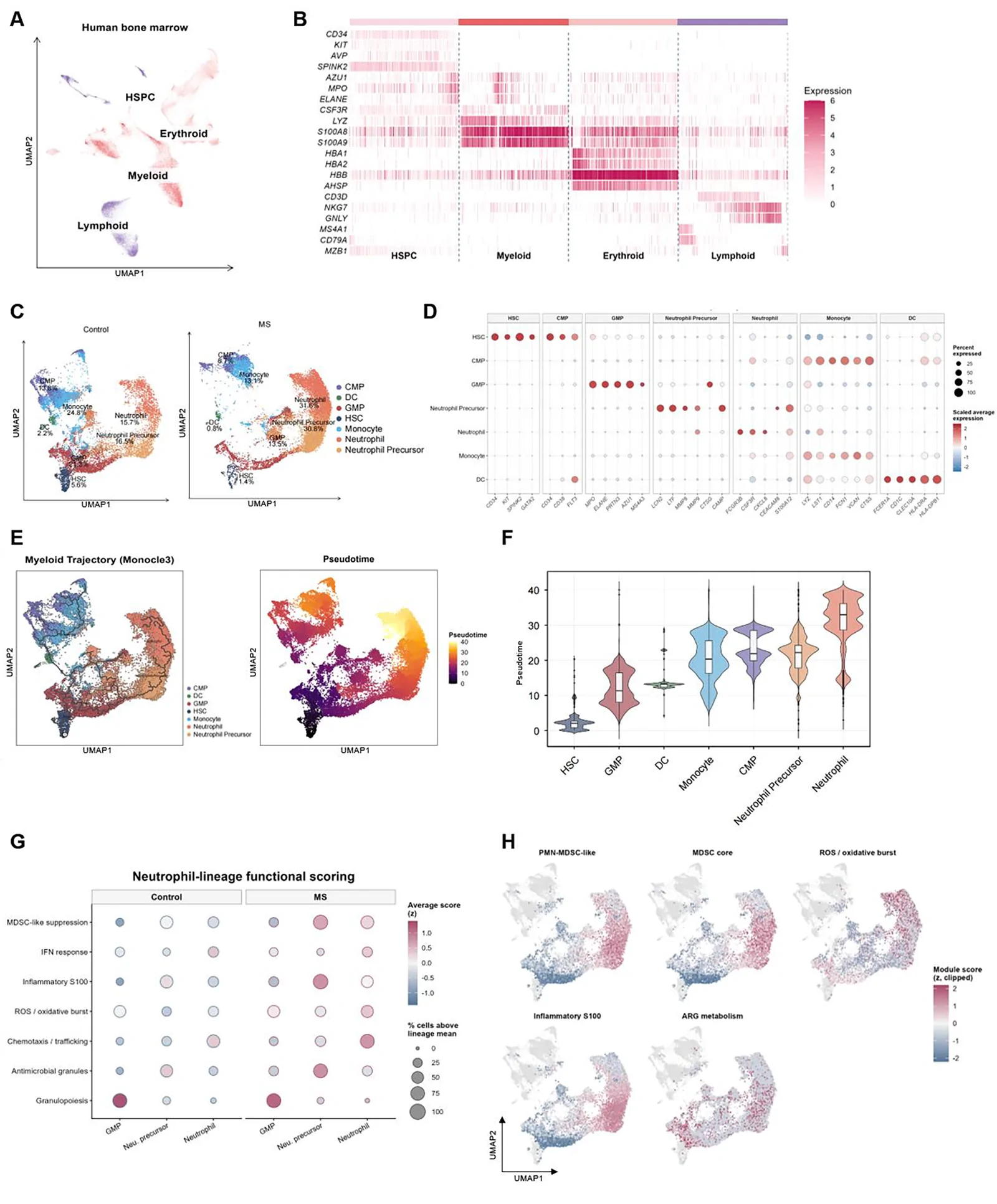
Myeloid skewing and neutrophil-lineage remodeling in MS bone marrow. **(A)** UMAP of human bone marrow single-cell transcriptomes showing HSPC, erythroid, myeloid, and lymphoid lineages. Each dot represents one cell and color denotes lineage. **(B)** Heatmap of representative marker genes for HSPC, myeloid, erythroid, and lymphoid/plasma cell lineages. Color indicates log-normalized expression. **(C)** UMAPs of control and MS myeloid cells showing HSC, CMP, GMP, neutrophil precursor, neutrophil, monocyte, and DC subsets with relative proportions. **(D)** Dot plot of myeloid subset markers. Dot size indicates the percentage of cells expressing each gene, and color indicates scaled average expression. **(E)** Monocle3 myeloid trajectory and pseudotime distribution. The trajectory was rooted in the HSC-enriched region and extended toward GMP, neutrophil precursor, and neutrophil states. **(F)** Violin plots of pseudotime distribution across myeloid subsets. **(G)** Functional module scores in GMPs, neutrophil precursors, and neutrophils from control and MS samples, including granulopoiesis, antimicrobial granules, chemotaxis/trafficking, ROS/oxidative burst, inflammatory S100, IFN response, and MDSC-like suppression. Dot size indicates the percentage of cells above the lineage mean, and color indicates average z-score. **(H)** UMAP distribution of PMN-MDSC-like, MDSC core, ROS/oxidative burst, inflammatory S100, and ARG metabolism modules.

Reclustering of the myeloid compartment resolved HSCs, common myeloid progenitors (CMPs), granulocyte-monocyte progenitors (GMPs), neutrophil precursors, mature neutrophils, monocytes, and dendritic cells (DCs) (**Figure 1C**). Relative to controls, the MS map showed greater representation of granulocytic regions, particularly neutrophil-precursor and neutrophil states, together with reduced representation of monocyte and early-progenitor regions. Marker expression substantiated this ordering: HSCs were enriched for CD34, KIT, and SPINK2; GMPs for MPO, ELANE, and PRTN3; neutrophil precursors and neutrophils for LCN2, LTF, MMP8/MMP9, CTSG, CEACAM8, FCGR3B, and CSF3R; monocytes for LYZ, LST1, FCN1, VCAN, and CTSS; and DCs for FCER1A, CLEC10A, and HLA-DRA (**Figure 1D**). The coordinated change across progenitor and mature granulocytic regions indicates that the MS-associated myeloid pattern involves the organization of the granulocytic continuum rather than a single terminal cluster.

We next reconstructed differentiation-associated variation with Monocle3 (15–17). With the HSC-enriched node designated as the root, pseudotime progressed through CMP/GMP states toward neutrophil precursors and mature neutrophils, while monocyte/DC branches remained distinct (**Figures 1E, F**). Functional scores were not distributed uniformly along this trajectory. PMN-MDSC-like, inflammatory-S100, ROS/oxidative-burst, and arginine-metabolism programs varied across the granulocytic continuum, with stronger signals in later granulocytic states (**Figures 1G, H**). These results link the PMN-MDSC-like program to a differentiation-associated transcriptional landscape that combines granule, inflammatory, oxidative, and amino-acid-metabolic features.

### PMN-MDSC-like states show heterogeneous granulocytic and ARG1-associated features

Within this myeloid landscape, we annotated M-MDSC-like and PMN-MDSC-like states (**Figure 2A**). M-MDSC-like cells retained a monocytic program characterized by CD14, LYZ, LST1, FCN1, VCAN, CCR2, and HLA-DRA. In contrast, PMN-MDSC-like cells combined a granulocytic backbone—S100A8, S100A9, S100A12, CSF3R, CXCR2, FCGR3B, CEACAM8, LCN2, and MMP9—with ARG1-associated effector genes including ARG1, PTGS2, CYBB, and NCF2 (**Figure 2B**). The distribution of these markers across cells also showed that the state was heterogeneous: granulocytic identity was broadly represented, whereas ARG1-associated effector expression varied within the compartment. This marker combination separated the RNA-resolved PMN-MDSC-like state from the monocyte-associated population and provided the basis for the subsequent pathway analyses.

**Figure 2:**
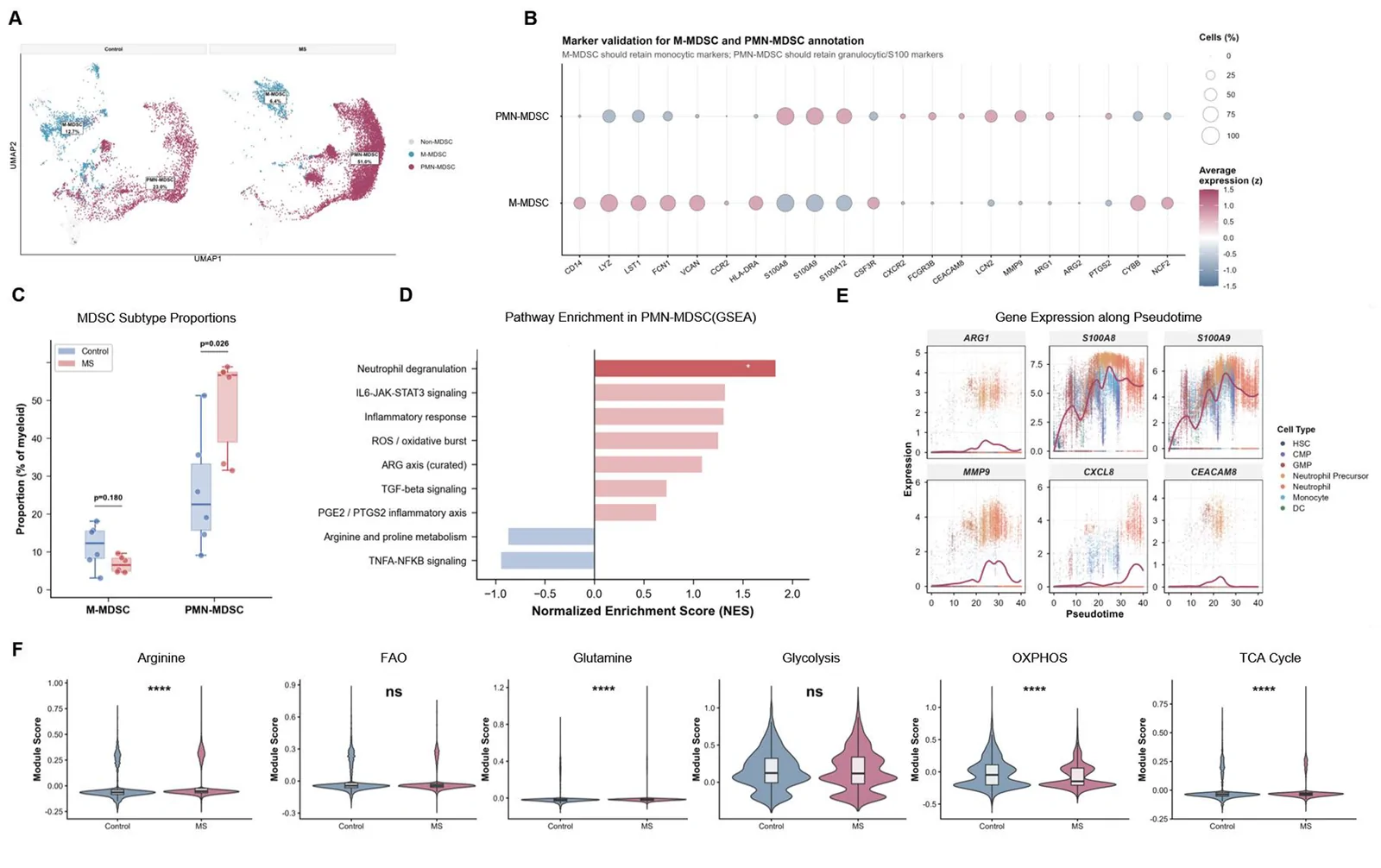
PMN-MDSC-like states and ARG1-associated pathway features in MS bone marrow. **(A)** UMAPs of control and MS myeloid cells annotated as M-MDSC-like, PMN-MDSC-like, or non-MDSC states; the in-panel MDSC labels are shorthand for these RNA-defined human states. **(B)** Marker validation of monocytic, granulocytic, and ARG1-associated genes. Dot size indicates the percentage of expressing cells and color indicates scaled average expression. **(C)** Sample-level subtype proportions; each point represents one donor and two-sided Wilcoxon P values are shown. **(D)** GSEA of PMN-MDSC-like pseudobulk profiles. Positive NES denotes enrichment in MS and negative NES denotes enrichment in controls; neutrophil degranulation was BH significant (NES = 1.83, FDR = 0.039). The in-panel label ‘ARG axis (curated)’ denotes the prespecified 12-gene ARG1-centered targeted module and is distinct from the database-defined arginine-and-proline metabolism pathway. **(E)** Expression patterns along inferred myeloid pseudotime. **(F)** Cell-level distributions of arginine, fatty-acid-oxidation, glutamine, glycolysis, oxidative-phosphorylation, and TCA-cycle scores; violin plots summarize cellular distributions and box plots show medians and quartiles. ****P≤0.0001; ns: not significant.

Sample-level analysis showed a higher PMN-MDSC-like proportion in MS (P = 0.026), whereas the M-MDSC-like proportion did not differ significantly (P = 0.180; two-sided Wilcoxon rank-sum tests; **Figure 2C**). The contrasting subtype results indicate that the change in the displayed analysis was concentrated in the granulocytic MDSC-like compartment rather than shared by both MDSC-like annotations. We therefore focused the next analyses on the transcriptional and metabolic organization of the PMN-MDSC-like state.

Pseudobulk GSEA further resolved the pathways associated with this state (**Figure 2D**). Neutrophil degranulation was the only BH-supported pathway (NES = 1.83, FDR = 0.039), with CD177, ITGB2, STXBP2, PRAM1, ITGAM, and VAMP2 in the leading edge. These genes span neutrophil activation, adhesion, vesicle docking, and granule exocytosis, linking the enrichment signal to a coherent secretory and trafficking program. The study-specific 12-gene set labeled in the retained panel as ‘ARG axis (curated)’ comprised ARG1, ARG2, NOS2, ASS1, ASL, ODC1, SAT1, SRM, AMD1, SLC7A1, SLC7A2, and SLC3A2. Functionally, this set connects arginine hydrolysis (ARG1 and ARG2), nitric-oxide substrate competition (NOS2), citrulline-to-arginine recycling (ASS1 and ASL), polyamine synthesis and turnover (ODC1, SAT1, SRM, and AMD1), and cationic amino-acid transport (SLC7A1, SLC7A2, and SLC3A2). ARG1, SAT1, and AMD1 formed the leading edge of this targeted module (NES = 1.09, FDR = 0.699). By comparison, the database-defined arginine-and-proline metabolism pathway covered a broader reaction space, including urea-cycle, glutamate/glutamine, proline, creatine, amine-oxidation, and aldehyde-dehydrogenase reactions; GLS formed its leading edge (NES=-0.87, FDR = 0.782). We therefore use ‘ARG1-centered targeted module’ for the study-specific gene set and reserve ‘arginine and proline metabolism’ for the database pathway. **Figure 2E** places individual ARG1-associated genes along inferred myeloid pseudotime, whereas **Figure 2F** shows the cell-level distributions of six metabolic programs, allowing the targeted ARG1 result to be interpreted within a broader metabolic context.

### Expression-based regulatory prioritization and co-expression analyses nominate an ARG1-associated human network

We then examined the regulatory organization of the ARG1-associated state. The cell-state heatmap positioned STAT3, CEBPB, HIF1A, CEBPA, SPI1, NFKB1, and IRF8 across distinct myeloid populations and showed that the PMN-MDSC-like compartment contained a composite rather than uniform TF-activity profile (**Figure 3A**). Cell-level UMAPs reinforced this point: STAT3, CEBPB, HIF1A, and CEBPA activities occupied partially overlapping but nonidentical regions, and TF-specific color ranges exposed within-TF spatial variation that was compressed on a common scale (**Figure 3B**). The MS-minus-control comparison in **Figure 3C** likewise showed mixed directions, including lower CEBPB and higher CEBPA activity estimates in MS. Thus, the displayed regulators do not behave as a single uniformly activated TF module; instead, they define a heterogeneous regulatory landscape around the ARG1-associated granulocytic state.

**Figure 3:**
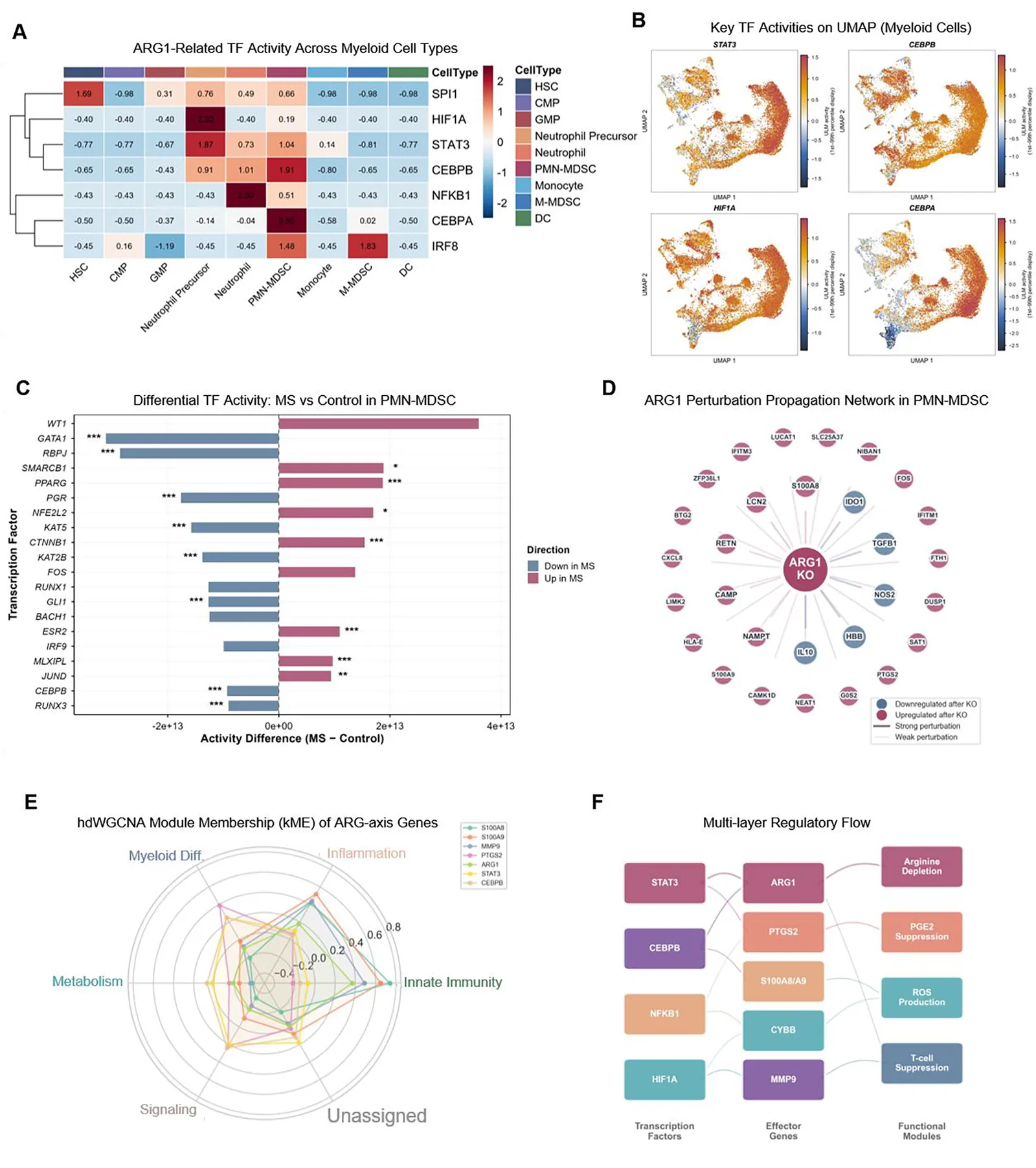
Human ARG1-associated transcriptional regulation and co-expression networks. **(A)** Heatmap of standardized ARG1-related TF activity across human myeloid cell states. **(B)** Cell-level UMAP distributions of STAT3, CEBPB, HIF1A, and CEBPA activity, displayed with TF-specific color ranges. **(C)** Cell-level comparison of TF activity in PMN-MDSC-like cells from MS and control samples; bars show MS-minus-control activity estimates and plotted significance annotations correspond to the displayed analysis. **(D)** Predicted propagation network after virtual ARG1 perturbation. **(E)** hdWGCNA module-membership (kME) profiles for ARG1-associated genes. **(F)** Multi-layer summary linking prioritized TFs, effector genes, and functional modules. *P < 0.05; **P ≤ 0.01; ***P ≤ 0.001.

Virtual ARG1 perturbation extended this analysis from TF activity to predicted downstream regulation. The propagation network in **Figure 3D** connected ARG1 perturbation to genes involved in inflammatory signaling, granule biology, metabolic control, and myeloid differentiation. In parallel, hdWGCNA placed ARG1, S100A8, S100A9, MMP9, PTGS2, STAT3, and CEBPB across functionally annotated co-expression modules (**Figure 3E**), showing that ARG1-associated genes participate in several coordinated transcriptional neighborhoods rather than a single isolated module. The multilayer summary in **Figure 3F** integrates candidate TFs, effector genes, and functional programs. Convergence among the TF maps, virtual perturbation, and co-expression structure prioritizes a connected ARG1-associated regulatory network for subsequent experimental testing.

### Inferred communication patterns and T-cell expression states

CellChat analysis placed PMN-MDSC-like cells among the prominent output populations in the bone-marrow communication network (**Figure 4A**). The MS-minus-control interaction heatmap showed that communication changes were distributed across several sender-receiver combinations rather than restricted to one pair (**Figure 4B**). Selected ligand-receptor probabilities and pathway-level information flow further resolved the signaling systems contributing to this network, including immune-recognition, adhesion, inflammatory, and growth-factor pathways (**Figures 4C, D**). Together, these panels identify the PMN-MDSC-like compartment as part of a broader remodeling of marrow intercellular communication.

**Figure 4:**
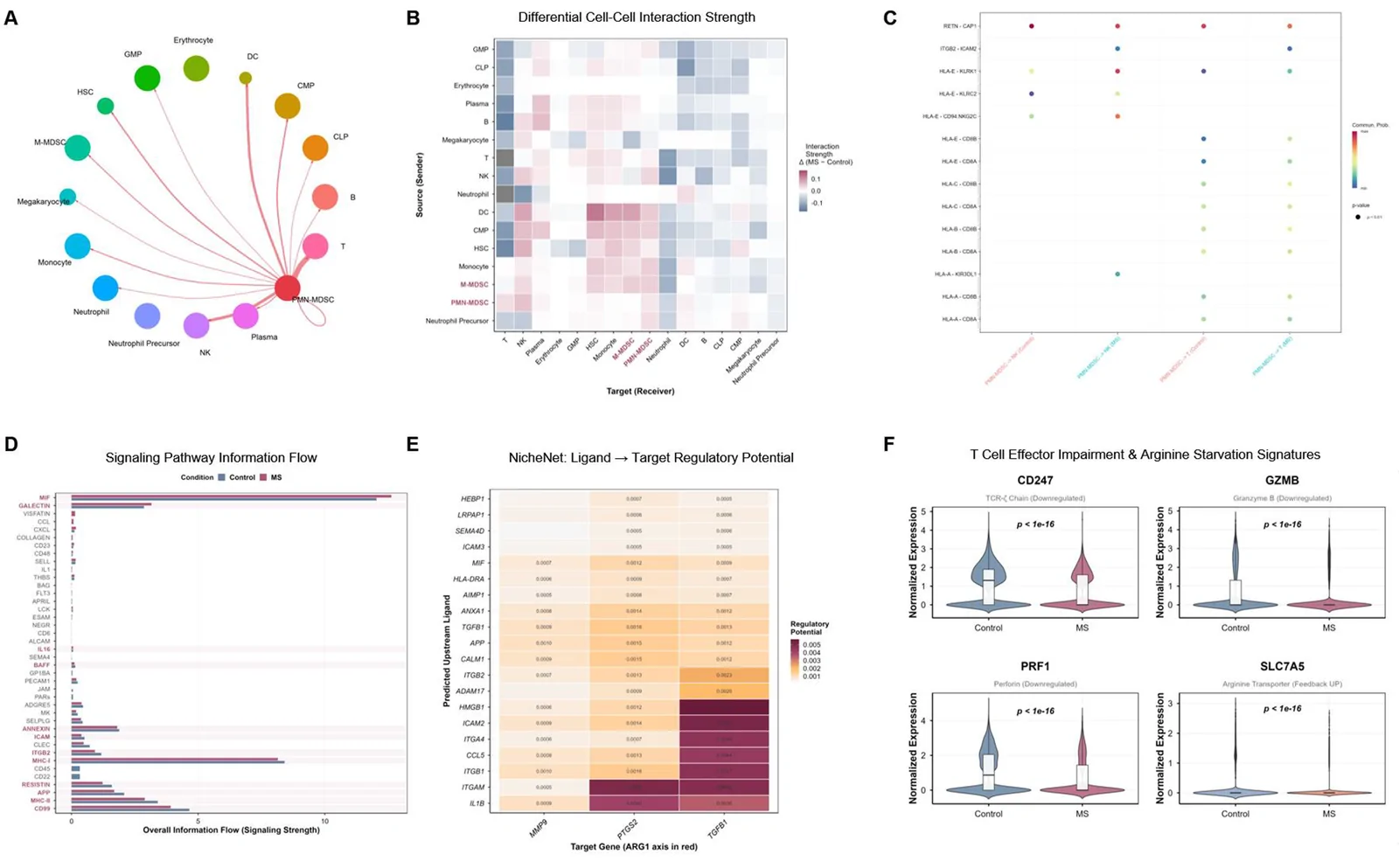
Inferred bone-marrow communication and T-cell expression patterns. **(A)** CellChat communication network centered on PMN-MDSC-like cells. **(B)** MS-minus-control differential interaction strength across sender and receiver populations. **(C)** Selected ligand-receptor interaction probabilities. **(D)** Signaling-pathway information flow in control and MS bone marrow. **(E)** NicheNet ligand-target regulatory-potential map for predicted upstream ligands from other bone-marrow myeloid populations and ARG1-associated target genes in PMN-MDSC-like receiver cells; rows are predicted ligands and columns are MMP9, PTGS2, and TGFB1. **(F)** Cell-level expression distributions of CD247, GZMB, PRF1, and SLC7A5 in control and MS T cells; all four genes were lower in MS in the displayed cell-level analysis. Donor-level module sensitivity analyses are reported separately in the text.

NicheNet was applied in the complementary direction to identify upstream signals associated with the PMN-MDSC-like ARG1 program. Other bone-marrow myeloid populations were treated as potential ligand sources, and PMN-MDSC-like cells as receivers. The retained regulatory-potential matrix links predicted upstream ligands to MMP9, PTGS2, and TGFB1 (**Figure 4E**). These target genes represent extracellular-matrix remodeling, prostaglandin-associated inflammatory regulation, and immunoregulatory signaling, respectively. The NicheNet result therefore refines the CellChat network by connecting the marrow signaling environment to specific components of the ARG1-associated receiver program.

We next examined T-cell expression states associated with this remodeled marrow environment. In the displayed cell-level analysis, CD247, GZMB, PRF1, and SLC7A5 were lower in MS T cells than in controls (all P<1×10^-16^; **Figure 4F**). CD247 reports TCR-complex integrity, GZMB and PRF1 represent cytotoxic effector machinery, and SLC7A5 is a major amino-acid transporter. These genes therefore capture complementary signaling, effector, and metabolic dimensions. In the separate donor-pseudobulk analysis, the composite TCR/effector module was comparable between groups (P = 0.383, FDR = 0.383), whereas the amino-acid-stress module was lower in MS (P = 0.0175, FDR = 0.035). The two analysis levels together indicate that the pronounced cell-level shifts coexist with a more selective donor-level module pattern, with the clearest donor-level difference involving amino-acid-stress responses.

### ARG1-associated computational analysis and EAE experimental validation

**Figure 5** consolidates the computational consequences of ARG1 perturbation. The full ranked differential-regulation view in **Figure 5A** shows the direction and magnitude of predicted gene responses, while **Figure 5B** summarizes the associated GO biological processes. These full views complement the focused network summaries in **Figures 3C, D, F** by retaining the broader ranked output. **Figure 5C** compares predefined reference perturbation patterns with selected virtual-knockout Z scores and produces a reflector score, whereas **Figure 5D** displays the underlying selected-gene template/effect matrix. Concordant and opposing response patterns can therefore be traced from their summary score back to the component genes. This layered presentation converts the virtual perturbation output into explicit regulatory and pharmacologic hypotheses for experimental prioritization.

**Figure 5:**
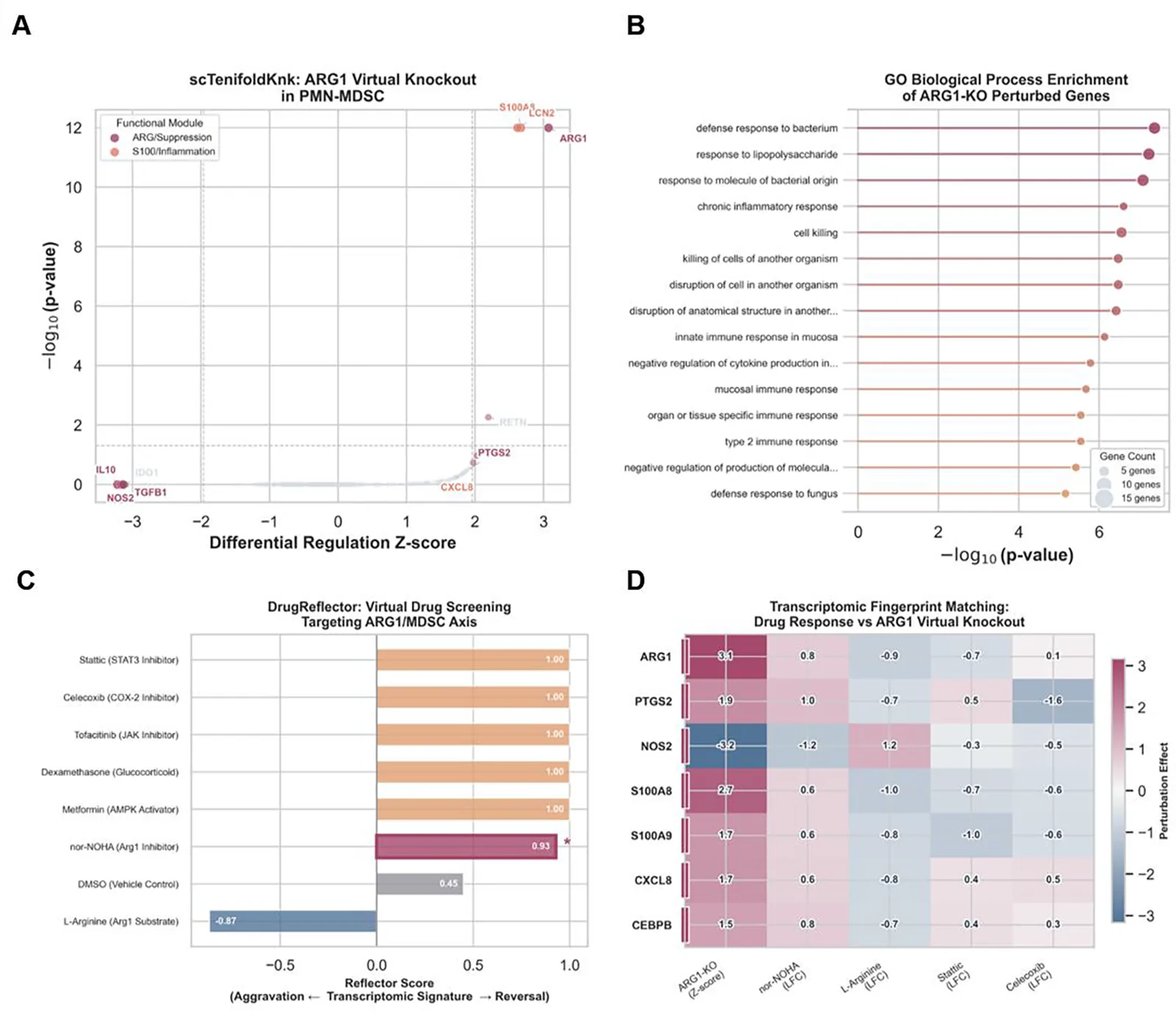
Computational prioritization of the ARG1-associated program. **(A)** Full virtual ARG1-perturbation differential-regulation view. **(B)** GO biological-process enrichment of predicted perturbed genes; the horizontal axis shows -log10(P value), as labeled in the retained panel. **(C)** Rule-based reference-pattern comparison using predefined perturbation templates and selected virtual-knockout Z scores. **(D)** Selected-gene template/effect fingerprint matrix. Together, the panels rank regulatory processes and concordant or opposing response patterns associated with ARG1 perturbation.

Finally, we examined the ARG1-associated pathway in EAE using a separate experimental sequence. Flow cytometry first gated live CD11b+ bone-marrow cells and then resolved Ly6G+Ly6Clo/neg PMN-MDSCs and Ly6C+ M-MDSCs (**Figure 6A**). EAE was accompanied by increased PMN-MDSC proportions and higher ARG1 mean fluorescence intensity within the gated PMN-MDSC population (**Figures 6B–D**), providing a protein-level counterpart to the ARG1-associated human transcriptional program. In the intervention experiment, oral nor-NOHA or vehicle was administered from day 1 after immunization. Nor-NOHA-treated mice showed lower clinical scores across the disease course and reduced demyelination and inflammatory infiltration in spinal-cord sections (**Figures 6E, F**). The aligned flow-cytometric, clinical, and histopathological readouts demonstrate that the arginase-associated program is pharmacologically responsive *in vivo* under the prophylactic dosing regimen used here.

**Figure 6:**
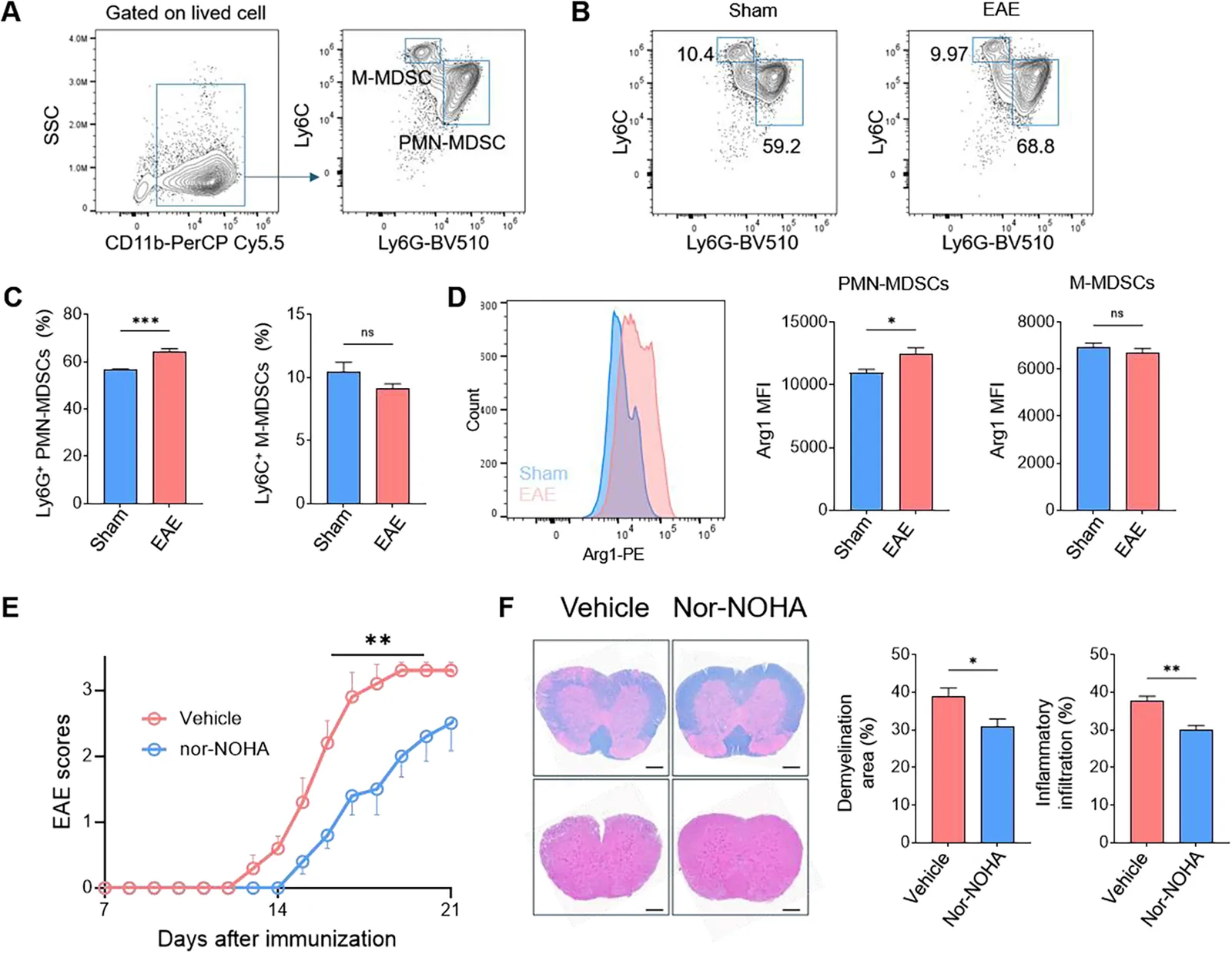
Cross-species EAE validation and systemic prophylactic arginase-pathway intervention. **(A)** Flow-cytometric gating of live CD11b+ bone-marrow cells into Ly6G+Ly6Clo/neg PMN-MDSCs and Ly6C+ M-MDSCs. **(B–D)** EAE-associated PMN-MDSC proportions and ARG1 protein measurements. **(E)** Clinical EAE scores after vehicle or nor-NOHA treatment begun on day 1 after immunization (n=8 per group); data are presented as mean ± SEM and were analyzed longitudinally as described in Methods. **(F)** Representative spinal-cord pathology and quantification; Luxol Fast Blue is shown in the upper row and H&E in the lower row, with vehicle and nor-NOHA columns and scale bars as shown. Data are presented as mean ± SEM; n = 8 per group. Two-group comparisons were analyzed using two-tailed unpaired t tests. *P< 0.05; **P< 0.01; ***P≤0.001.

## Discussion

This study integrates human bone-marrow single-cell analysis with EAE experiments to define an ARG1-associated immunometabolic program in PMN-MDSC-like cells. The human analysis resolves three connected levels of organization: granulocytic differentiation, an ARG1-associated regulatory and metabolic network, and altered marrow communication accompanied by a T-cell amino-acid-response phenotype. The mouse experiments add ARG1 protein measurements and a systemic prophylactic pharmacologic response. Considered together, these findings position the ARG1-associated granulocytic program at the intersection of marrow myelopoiesis, immune-cell communication, T-cell metabolism, and neuroinflammation.

The first important feature is that the PMN-MDSC-like state is embedded within granulocytic remodeling. The trajectory and module-score analyses place PMN-MDSC-like, inflammatory-S100, ROS/oxidative-burst, and arginine-metabolism features along the neutrophil differentiation continuum rather than in an isolated cell island. This organization is consistent with the strong neutrophil-degranulation enrichment and its leading-edge genes involved in adhesion and vesicle release. At the same time, the marker and TF maps show substantial within-state heterogeneity. The combination of a shared granulocytic backbone with variable ARG1-associated effectors and nonuniform TF activity suggests that the biologically relevant unit is a spectrum of granulocytic states with differing regulatory and metabolic intensity.

A second feature is the distinction between the focused ARG1-centered module and the broader arginine-and-proline metabolism pathway. The targeted module was constructed to capture arginine consumption, recycling, transport, nitric-oxide competition, and polyamine production, whereas the database pathway includes a wider set of reactions extending into proline, glutamate/glutamine, creatine, and aldehyde metabolism. Their different gene membership, leading edges, and enrichment directions explain why the two labels are not interchangeable. This distinction also helps interpret the T-cell findings. L-arginine availability can influence CD3-zeta expression and T-cell proliferation in experimental systems (18, 19), and the present marrow data show coordinated changes in CD247, cytotoxic-effector genes, SLC7A5, and the donor-level amino-acid-stress module. The results therefore connect the myeloid ARG1-associated program with a specific amino-acid-response context in T cells.

The regulatory analyses provide a third layer of interpretation. STAT3, CEBPB, HIF1A, CEBPA, SPI1, NFKB1, and IRF8 displayed state-dependent and spatially heterogeneous activity rather than a common upward shift. Virtual ARG1 perturbation and hdWGCNA then linked these candidate regulators to ARG1, S100-family genes, MMP9, PTGS2, and broader inflammatory and metabolic modules. CellChat and NicheNet extended the network beyond the PMN-MDSC-like compartment by identifying candidate intercellular inputs associated with MMP9, PTGS2, and TGFB1. The convergence of intracellular regulation, co-expression, and ligand-target structure provides a coherent framework for selecting perturbations and readouts in subsequent mechanistic experiments. Matched chromatin-accessibility and motif analyses in MS and control marrow would further resolve which expression-based TF priorities are supported at the regulatory-element level.

The EAE experiments place the human transcriptional findings in an *in vivo* inflammatory setting. Increased ARG1 protein within flow-gated bone-marrow PMN-MDSCs parallels the ARG1-associated granulocytic program identified in human marrow, while nor-NOHA treatment was accompanied by improved clinical and spinal-cord pathological readouts. The agreement among flow-cytometric, longitudinal clinical, and histological measures strengthens the interpretation that the arginase-associated pathway is biologically active and pharmacologically responsive in this model. Because the intervention began soon after immunization, the current experiment most directly informs early systemic pathway modulation. Cell-resolved perturbation, exposure-response measurements, and post-onset dosing can now define the responsible cellular contributors and the timing of maximal response.

These findings also refine the context-dependent literature on MDSCs in MS and EAE. Prior studies showed that miR-223 regulates MDSC abundance and function and that MDSCs from patients with MS can display altered STAT3/ARG1 expression and suppressive activity (12, 13). In experimental tolerance models, PMN-MDSCs or antigen-expressing MDSCs suppress CD4+ T-cell proliferation and ameliorate EAE, including ARG1-dependent settings (10, 11). The present study adds a bone-marrow-resolved human program, separates the granulocytic PMN-MDSC-like state from the monocyte-associated state, and connects it to an ARG1-responsive EAE phenotype. This combination helps explain why MDSC behavior may differ according to tissue compartment, differentiation stage, inflammatory context, and the balance among granulocytic, metabolic, and regulatory programs.

The marrow ARG1-associated program has practical implications for biomarker development and experimental design. Fresh paired blood and marrow samples would allow direct comparison of PMN-MDSC abundance, ARG1 protein or enzymatic activity, and ex vivo T-cell suppression or rescue in the same patients. Incorporating sample-processing variables, standardized whole-blood and phenotypic workflows, and complementary markers such as LOX-1 would improve phenotypic resolution (20–23). Larger cohorts and longitudinal sampling could then determine whether the marrow program is represented in blood and how it tracks clinical activity, treatment response, and disability accumulation. In parallel, conditional Arg1 perturbation in defined myeloid lineages would connect the human transcriptional state, the inferred regulatory network, and the *in vivo* pharmacologic phenotype at cellular resolution.

In summary, the study identifies an ARG1-associated PMN-MDSC-like transcriptional program within granulocytic remodeling in MS bone marrow and links it to altered intercellular communication and T-cell amino-acid-response features. EAE experiments show increased PMN-MDSCs and ARG1 protein and demonstrate a clinical and pathological response to systemic prophylactic arginase-pathway inhibition. These findings establish an integrated peripheral immunometabolic axis spanning marrow differentiation, ARG1-associated regulation, T-cell metabolic state, and neuroinflammatory disease. Conditional Arg1 perturbation, paired functional assays, and post-onset intervention studies provide a direct route for developing this mechanistic framework.

## Methods

### Human bone marrow samples and data source

Bone marrow single-cell RNA-seq data were obtained from Shi et al. (8). The original study included seven treatment-naive patients with MS (two male and five female) and seven healthy controls (two male and five female) for single-cell RNA-seq. Age did not differ significantly between MS and control groups (33.7 ± 12.8 vs 34.4 ± 17.5 years, P = 0.932). Bone marrow flow cytometry validation in the source study included 14 treatment-naive patients with MS and 14 healthy individuals. Inclusion criteria, exclusion criteria, ethics approval, and informed consent followed the original study. The present work is a secondary reanalysis and mechanistic extension using the dataset deposited under GSA HRA001783.

### Single-cell RNA-seq preprocessing

The source data were generated with the 10x Chromium platform and aligned to human GRCh38, Ensembl 98, using Cell Ranger v5.0.0 (8). Following the original Cell study, `--force-cell` and `--intron-include` were used to enrich neutrophil capture. The original quality-control thresholds removed cells with fewer than 180 detected genes or mitochondrial transcript fraction greater than 10% (percent.mt > 10%).

For the present reanalysis, we applied a stricter lower gene threshold while retaining high mitochondrial fractions that may occur in neutrophil-lineage cells: each cell was required to have at least 200 detected genes, no more than 8,000 detected genes, mitochondrial transcript fraction no higher than 20%, and each retained gene had to be expressed in at least three cells. Expression matrices were log-normalized in Seurat with a scale factor of 10,000; 2,000 highly variable genes were selected; mitochondrial percentage was regressed during scaling; up to 50 principal components were computed; global graph clustering used resolution 0.5; and myeloid reclustering used resolution 0.6. Batch correction was performed with Harmony and low-dimensional visualization with UMAP (24–30).

### Batch correction, clustering, and cell annotation

The original study used Seurat v3.2.0 and Harmony v1.0 for sample batch correction, selected the first 15 PCs explaining more than 80% of total variance, and used Louvain/SLM graph clustering at resolution 0.5 followed by 1,000 force-directed graph iterations for global annotation (8, 25, 28, 31, 32). In the present reanalysis, broad annotations were retained and myeloid cells were reclustered for secondary annotation. Cell identities were assigned using canonical markers: HSPCs (CD34, KIT, SPINK2), myeloid cells (MPO, ELANE, CSF3R, LYZ, S100A8, S100A9), erythroid cells (HBA1, HBA2, HBB, AHSP), and T/NK/B/plasma cells (CD3D, NKG7, GNLY, MS4A1, CD79A, MZB1). Myeloid cells were further annotated as HSCs, CMPs, GMPs, neutrophil precursors, neutrophils, monocytes, and DCs.

### MDSC-like subtype annotation and donor-level proportion analysis

For human RNA-seq analyses, ‘PMN-MDSC-like’ denotes a transcriptomically annotated granulocytic state and ‘M-MDSC-like’ a monocyte-associated state. Annotation combined a granulocytic backbone with ARG1-associated effector genes and explicit separation from monocyte markers. This transcriptomic definition supports consistent state mapping across donors and complements morphology, protein phenotyping, ARG1 activity, and T-cell suppression/rescue assays. In mouse flow cytometry, Ly6G+Ly6Clo/neg cells are termed PMN-MDSCs according to the gated experimental definition.


$$P_{s,k}=100\times \frac{N_{s,k}}{N_{s,myeloid}}$$

where N_s,k is the number of cells in subset k from sample s, and N_s,myeloid is the total number of myeloid cells from the same sample. Sample-level proportions were compared between control and MS groups using two-sided Wilcoxon rank-sum tests, with donor as the unit of analysis.

### Trajectory inference

Monocle3 and SeuratWrappers were used to convert the Seurat object into a cell_data_set (15–17). Seurat UMAP coordinates were retained. After `cluster_cells`, the trajectory graph was learned using `learn_graph(use_partition=TRUE)`. The principal graph node most enriched for HSCs was used as the root, and pseudotime was computed with `order_cells(root_pr_nodes=root_node)`. Pseudotime expression of ARG1, S100A8, S100A9, MMP9, CXCL8, and CEACAM8 was visualized with single-cell scatter plots and loess smoothing.

### Module scoring, pseudobulk GSEA, and metabolic pathway analysis

Neutrophil-lineage functional modules included granulopoiesis (MPO, ELANE, PRTN3, AZU1, CTSG, MS4A3, CEBPE), antimicrobial granules (LCN2, LTF, MMP8, MMP9, CAMP, CEACAM8, FCGR3B, CSF3R), chemotaxis/trafficking (CXCR2, CXCR4, FPR1, FPR2, C5AR1, LTB4R, CXCL8, SELL, ITGAM), ROS/oxidative burst (CYBB, NCF1, NCF2, NCF4, RAC2, MPO, SOD2, GPX1), inflammatory S100 (S100A8, S100A9, S100A12, IL1B, LCN2), IFN response (ISG15, IFIT1, IFIT2, IFIT3, MX1, OAS1, IFI6, IRF7, STAT1), and MDSC-like suppression (ARG1, ARG2, PTGS2, CYBB, S100A8, S100A9, VEGFA, IL10, TGFB1, CD274, LGALS9). Module scoring followed the rationale of single-cell gene-set scoring. For cell c, module M, and detectable genes G_M, the raw score was:


$$S_{c,M}=\left(\frac{1}{\left|G_{M}\right|}\right)\sum_{\left(g\in G_{M}\right)E_{g,c}}$$

where E_g,c is log-normalized expression. Scores were then standardized within neutrophil-lineage cells:


$$Z_{c,M}=\frac{S_{c,M}-\mu_{M}}{\sigma_{M}+\epsilon }$$

where μ_M and σ_M are the mean and standard deviation for module M, and ϵ=10^-8 avoids division by zero. In dot plots, color represents the group or cell-type mean of the standardized score, and dot size represents the percentage of cells with a positive standardized score.

For GSEA, each donor contributed one PMN-MDSC-like pseudobulk profile. The prespecified ARG1-centered targeted module comprised ARG1, ARG2, NOS2, ASS1, ASL, ODC1, SAT1, SRM, AMD1, SLC7A1, SLC7A2, and SLC3A2 and was analyzed separately from the database-defined KEGG arginine-and-proline metabolism pathway obtained through MSigDB (33, 34). Normalized enrichment scores and BH-adjusted FDR values are reported for each pathway.

edgeR used TMM normalization, quasi-likelihood negative binomial generalized linear models, and robust dispersion estimation. Target-cell pseudobulk comparisons required at least 20 target cells per sample. GSEA used pseudobulk logFC as the ranking statistic, with positive values indicating higher expression in MS. Standard GSEA parameters were minSize=10 and maxSize=500; prespecified targeted-module analysis used minSize=3, maxSize=500, and eps=0. Gene sets included the ARG1-centered targeted module, IL6-JAK-STAT3, TGF-beta, PTGS2/PGE2, ROS/oxidative burst, neutrophil degranulation, TNFA-NFKB, and arginine/proline metabolism.

### Metabolic and T-cell functional readouts

**Figure 2F** reports cell-level module-score distributions in PMN-MDSC-like cells for arginine metabolism, fatty-acid oxidation, glutamine metabolism, glycolysis, oxidative phosphorylation, and the TCA cycle. For a cell c, pathway M, and detectable genes G_M, the score was the mean log-normalized expression of the pathway genes and was standardized within PMN-MDSC-like cells. The displayed control and MS distributions contain 3,130 and 8,310 cells, respectively, and were compared with two-sided cell-level Wilcoxon tests with BH adjustment across the six pathways.

### T-cell expression and donor-level module readouts

For **Figure 4F**, log-normalized cell-level expression of CD247, GZMB, PRF1, and SLC7A5 was compared between control and MS T cells using two-sided Wilcoxon tests with BH adjustment across the displayed genes. Donor-pseudobulk modules were computed separately. The TCR/effector module comprised CD247, CD3D, CD3E, CD3G, LCK, ZAP70, LAT, GZMB, PRF1, and IFNG; the amino-acid-stress module comprised ATF4, DDIT3, EIF2AK4, ASNS, SLC7A5, SLC7A1, and SLC3A2. Each donor contributed one module score, and donor-level comparisons used two-sided Wilcoxon rank-sum tests with BH correction across the two modules.

### Expression-based transcription-factor prioritization and co-expression network analysis

Expression-based TF activity was inferred with decoupleR using CollecTRI regulons retrieved through OmniPath (35–38). Regulons required at least five detectable target genes. **Figure 3A** summarizes standardized TF activities across annotated myeloid cell states. **Figure 3B** projects the resulting cell-level activities onto the myeloid UMAP using a separate color range for each TF. For **Figure 3C**, MS-versus-control differences were calculated in PMN-MDSC-like cells and displayed as exploratory cell-level activity contrasts with BH-adjusted Wilcoxon results. A donor-pseudobulk TF analysis across all 14 donors was also performed as a sensitivity analysis; no displayed TF survived BH correction at the donor level.

The cell-level and donor-pseudobulk TF analyses were interpreted jointly: the UMAP and cell-state heatmap define the spatial and lineage distribution of activity, the **Figure 3C** comparison summarizes the displayed group contrast, and the donor-pseudobulk analysis evaluates the stability of group effects across independent samples.

Co-expression network analysis was performed with WGCNA and hdWGCNA (39–41). Genes detected in at least 5% of cells were eligible. Metacells were generated by final cell type and condition with k=25 nearest neighbors and maximum shared overlap of 10. A signed network was constructed, modules were summarized by module eigengenes, and gene-module membership was calculated as the correlation between gene expression and module eigengene.

ARG1-associated genes (ARG1, S100A8, S100A9, MMP9, PTGS2, STAT3, and CEBPB) were visualized by module membership across co-expression modules.

### ARG1 virtual knockout and signature reversal scoring

ARG1 virtual knockout was performed in human MS PMN-MDSC-like cells using scTenifoldKnk (42). Cells were downsampled to a maximum of 1,000. Genes expressed in more than 5% of cells were retained, and 2,000 highly variable genes plus ARG1/PTGS2/NOS2/STAT3/CEBPB/IDO1/S100A8/S100A9/IL10/TGFB1 were forced into the target set. Up to 1,200 genes were used for network construction. The virtual knockout used 10 network replicates and 500 cells per replicate. Differential regulation was summarized as a Z-score:


$$Z_{g}=\frac{\Delta_{g}-\mu_{\Delta }}{\sigma_{\Delta }+\epsilon }$$

where Δ_g is the perturbation-induced regulatory shift. Two-sided p values were computed from the standard normal distribution and adjusted by the BH method. Significant perturbed genes were defined by |Z_g|>1.96 with adjusted p value criteria where applicable. GO biological process enrichment was performed with clusterProfiler.

Rule-based reference-pattern comparisons used predefined perturbation templates against virtual-knockout Z scores for selected genes. The reflector score quantified concordance between each reference pattern and the ARG1 virtual-knockout response. **Figure 5D** shows the selected-gene template/effect matrix used to construct the response fingerprint.

Positive scores indicate alignment with a recovery-like signature, whereas negative scores indicate alignment with the pathogenic direction. The score ranks reference patterns according to concordance with the ARG1 virtual-knockout response.

### Cell-cell communication and ligand-target modeling

Cell-cell communication was inferred with CellChat (43). Major cell types were used as sender and receiver groups. Differential interaction strength was calculated as MS-minus-control communication strength for each source-receiver pair.

Pathway-level information flow was compared between conditions. Candidate pathways included GALECTIN, SPP1, PVR, MIF, RESISTIN, TGF-beta, MHC-I, MHC-II, APP, ICAM, ITGB2, and CD99.

NicheNet was used to link ligands expressed by DCs, monocytes, GMPs, HSCs, CMPs, neutrophils, neutrophil precursors, and M-MDSC-like cells to an ARG1-associated response program in PMN-MDSC-like receiver cells (44). Receiver genes included ARG1/ARG2/ASS1/ASL/OAT/ODC1 together with S100A8, S100A9, MMP9, CYBB, PTGS2, NOS2, IDO1, IL10, TGFB1, VEGFA, CD274, LGALS9, NT5E, OLR1, and LCN2 when detectable. **Figure 4E** shows the retained ligand-target regulatory-potential matrix for MMP9, PTGS2, and TGFB1; rows are predicted upstream ligands and columns are PMN-MDSC-like target genes.

### EAE induction and clinical scoring

EAE induction followed the Cell study by Shi et al. and standard MOG35–55 EAE protocols (8, 45–48). Eight-week-old C57BL/6J mice were used for active immunization and randomized to vehicle or nor-NOHA treatment groups (n=8 per group). Mice were immunized subcutaneously at upper and lower back sites with an emulsion containing 200 μg MOG35–55 peptide in complete Freund’s adjuvant containing 500 μg nonviable desiccated Mycobacterium tuberculosis at 10 mg/mL. Pertussis toxin (200 ng) was administered intraperitoneally 2 h after immunization and again on day 2. Body weight and neurological scores were recorded daily by an investigator blinded to treatment group. Scores ranged from 0 to 5 in 0.5 increments: 0, no signs; 1, complete limp tail; 2, hindlimb weakness; 3, hindlimb paralysis; 4, forelimb paralysis; and 5, moribund.

### Nor-NOHA intervention

EAE mice were randomized to vehicle or nor-NOHA groups (n=8 per group). Nor-NOHA acetate (Nω-hydroxy-nor-L-arginine acetate; MedChemExpress/MCE, Cat# HY-112885A; CAS 1140844-63-8) was dissolved in sterile 0.9% NaCl and administered by oral gavage at 40 mg/kg. Vehicle mice received the same volume of 0.9% NaCl. Treatment was given every other day from Day 1 to Day 21 after immunization, corresponding to Day 1, 3, 5, 7, 9, 11, 13, 15, 17, 19, and 21 (11 doses total). Using a standard gavage volume of 10 mL/kg (0.1 mL/10 g), the working concentration was 4 mg/mL:

For a 20 g mouse, the gavage volume was 0.2 mL and the dose was 0.8 mg per administration. For n=8 mice, each dosing day required 1.6 mL working solution containing 6.4 mg nor-NOHA acetate; 1.8-2.0 mL was prepared to allow 10-20% excess. This 4 mg/mL working solution corresponds to approximately 13.5 mM based on molecular weight 296.28 g/mol. A commercial 10 mM in water format corresponds to approximately 2.96 mg/mL and would deliver approximately 29.6 mg/kg at 10 mL/kg; therefore, the 40 mg/kg regimen used a 4 mg/mL working solution or required volume adjustment. The 40 mg/kg oral dose was based on mouse oral gavage studies in which nor-NOHA was dissolved in 0.9% NaCl and administered at 40 mg/kg (49). Inflammatory and vascular disease models also commonly use 40 mg/kg/day intraperitoneally, and this dose can reduce ARG1/ARG2 activity with acceptable tolerability (50). Given the relatively rapid clearance of nor-NOHA, pharmacodynamic interpretation was based on ARG1 MFI, histopathology, and, where available, arginase activity or arginine pathway readouts (51).

### Mouse bone marrow flow cytometry and ARG1 intracellular staining

For terminal tissue collection, mice were deeply anesthetized with tribromoethanol (250 mg/kg, intraperitoneally). Adequate anesthetic depth was confirmed by loss of the pedal withdrawal reflex. Mice were then euthanized by cervical dislocation under deep anesthesia, and death was confirmed by cessation of respiration and heartbeat before femoral bone marrow and spinal cord tissues were collected. Bone marrow flow cytometry followed the Cell study and immunological flow cytometry guidelines (52, 53). Femoral bone marrow was flushed with cold PBS and filtered through a 70 μm cell strainer. Red blood cells were lysed with ACK lysis buffer, and cells were washed and resuspended in 1% BSA. Fluorescent antibodies were incubated on ice for 30 min. Intracellular ARG1 staining was performed after surface staining: cells were fixed for 20 min, washed twice with permeabilization buffer, stained with ARG1-PE antibody for 45 min, washed, and resuspended in flow buffer. Data were acquired on a FACSAria III or equivalent cytometer and analyzed with FlowJo v10.

The gating strategy was: live single cells -> CD11b+ myeloid cells -> Ly6G+Ly6Clo/neg PMN-MDSCs and Ly6G-Ly6Chi M-MDSCs -> ARG1-PE MFI/%positive. Channels shown in **Figure 6** include CD11b-PerCP-Cy5.5, Ly6G-BV510, Ly6C, and intracellular ARG1-PE. The figure documents the gating hierarchy and measured channels; no additional channel assignments are inferred here.

### Histology and pathology quantification

Spinal cord H&E staining was used to assess inflammatory infiltration, and Luxol Fast Blue staining was used to assess demyelination (8, 45, 54). Following the Cell study, spinal cord sections were deparaffinized, rehydrated, stained with 0.1% Luxol Fast Blue at 60 °C for 8 h, rinsed in distilled water, differentiated in 95% ethanol until gray-white matter boundaries were clear, dehydrated, and mounted. Images were acquired by light microscopy. Demyelinated area and inflammatory infiltration were quantified with ImageJ/Fiji (54). Quantification was performed by investigators blinded to treatment group.

### Statistical analysis

Statistical analyses were performed in R and GraphPad Prism. Two-group comparisons used two-tailed unpaired Student’s t tests or Mann-Whitney/Wilcoxon rank-sum tests depending on distribution and sample size. Multiple-group comparisons used one-way ANOVA with Tukey *post hoc* testing. Longitudinal EAE clinical scores were analyzed using two-way repeated-measures ANOVA or linear mixed models, reporting group, time, and interaction effects. Multiple testing was controlled by the Benjamini-Hochberg false discovery rate method. Animal scoring, pathology quantification, and flow cytometry analysis were performed with blinding whenever possible. Results are presented as mean ± SEM or median with interquartile range, and exact P values are reported where available.

## Data Availability

The original contributions presented in the study are included in the article/supplementary material. Further inquiries can be directed to the corresponding authors.
